# Quality-Gated Circularity Assessment of PET, Aluminium, and Reusable Glass Packaging in Deposit Return Systems

**DOI:** 10.3390/ma19173669

**Published:** 2026-08-28

**Authors:** Olga Orynycz, Jonas Matijošius, Andrzej Wasiak, Marta Wakulewska, Michał Sąsiadek

**Affiliations:** 1 Department of Production Management, Faculty of Engineering Management, Bialystok University of Technology, Wiejska Street 45A, 15-351 Bialystok, Poland; 2 Mechanical Science Institute, Vilnius Gediminas Technical University-VILNIUS TECH, Plytinės Str. 25, LT-10105 Vilnius, Lithuania; 3 Department of Social and Technical Sciences, University College of Professional Education, Plac Powstańców Śląskich 1/201, 53-329 Wrocław, Poland; andrzej.wasiak@wskz.pl; 4 Faculty of Engineering Management, Bialystok University of Technology, Wiejska Street 45A, 15-351 Bialystok, Poland; marta.wakulewska@gmail.com; 5 Institute of Mechanical Engineering, University of Zielona Gora, 65-516 Zielona Gora, Poland; m.sasiadek@iim.uz.zgora.pl

**Keywords:** PET, rPET, aluminium can, reusable glass, material circularity, quality gate, food-contact recycling, closed-loop recycling, material-flow analysis

## Abstract

**Highlights:**

- A quality-gated model separates return rate, material quality and processing yield.
- Pilot PET data show regional differences in purity, colour and washed-flake yield.
- Lithuania shows higher PET quality than the Polish regional average.
- The HQR index supports material-specific DRS circularity reporting.
- Smart-village information tools may improve rural DRS material quality.

**Abstract:**

Deposit return systems (DRS) can increase the capture of beverage packaging, but material circularity is not determined by return rate alone. A returned container contributes to high-value circularity only if it passes recognition, sorting, pre-processing, and material-specific quality gates. This article evaluates the material-quality performance of three returned beverage-packaging materials—polyethylene terephthalate (PET), aluminium and reusable glass—using a quality-gated high-value recovery framework. The model defines a high-quality recovery index, *HQR* = *R* × *Q* × *Y*, where R is the return rate, Q is the quality factor of the returned stream, and Y is the reprocessing or reuse yield. The *HQR* indicator describes the quality of the entire DRS process. The core purpose of the *HQR* model is to distinguish nominal packaging return from high-quality material recovery and to show whether returned PET, aluminium and reusable glass streams remain suitable for high-value circular pathways. A survey-supported early-stage return scenario (*R* = 0.50) is compared with the 77% and 90% separate-collection targets used in European policy. To strengthen the PET branch of the model, a pilot PET stream-quality and processing-yield dataset was incorporated, including PET purity, colour composition, non-PET impurities, residual moisture, organic residues, intrinsic viscosity, washed PET flake or pellet yield, and sorting/washing rejection. The pilot data indicate that Lithuania had higher PET quality (98.2% PET purity, 80% clear PET, 1.8% non-PET impurities, IV = 0.74 dL/g, and 84.5% washed PET yield) than the Polish regional average (94.3% PET purity, 72.7% clear PET, 5.7% non-PET impurities, IV = 0.721 dL/g, and 80.3% washed PET yield). At *R* = 0.50, the pilot-derived PET HQR is approximately 37.8% for Lithuania and 31.6% for the Polish regional average. The results indicate that the same nominal return rate can lead to substantially different high-quality recovery outcomes because PET is constrained by stream purity, colour, contamination, and processing yield; aluminium by alloy and remelting control; and reusable glass by inspection, breakage, and refill compatibility. The proposed framework can support structured DRS operator reporting by identifying the material-quality and yield variables that should be measured alongside mass collection; however, operator-level validation is required before the model can be used as a predictive performance tool.

## 1. Introduction

Beverage packaging is a useful test case for circular material management because the three dominant container types do not behave as one material family. PET bottles are polymer products whose bottle-to-bottle value depends on sorting accuracy, colour, contamination control, decontamination, and food-contact approval. Aluminium cans enter a metal-recycling route in which scrap purity, alloy compatibility, and remitting performance determine whether a can-to-can pathway is realistic. Refillable glass bottles follow a product-retention route: the bottle must be returned, inspected, washed, and kept in a refill pool before it can deliver further rotations.

The focus of this study is therefore the material-quality performance of returned packaging materials, not the organisational performance of the deposit system itself. The deposit return system is treated only as a source of separated PET, aluminium and reusable glass streams. The central question is whether these materials retain the physical, chemical and functional quality required for high-value circular pathways.

Policy setting has made these substantive distinctions practically significant. The Single-Use Plastics Directive sets ambitious targets for the separate collection of plastic beverage bottles (77% by 2025 and 90% by 2029) and also establishes recycled-content obligations for PET as well as other beverage bottles [1,2]. The Packaging and Packaging Waste Regulation (EU) 2025/40 goes farther in this direction, setting rules on recyclability, recyclable content, reuse, and deposit-return schemes [3]. Since 1 October 2025, Poland, has implemented a deposit-refund system for single-use plastic bottles up to 3 L, metal cans up to 1 L, and reusable glass bottles up to 1.5 L [4].

And size matters in terms of the quality of these flows. In 2023, EU countries generated 79.7 million tons of packaging garbage, or 177.8 kg per person. Plastics, glass, and metals all played a part in this total [5]. A deposit system can help segregate beverage containers from mixed municipal waste and aid in their recognition and tracking. But just because a container is collected via a deposit channel does not mean it will become food-grade rPET, can-to-can aluminium, or a refillable glass bottle held for another use cycle.

For this reason, material assessments have to keep apart three levels that are often blurred in policy discussion: the number or mass of items returned, the fraction that remains technically suitable for a high-value route, and the amount that finally leaves the process as a secondary material or retained reusable product. The distinction is particularly sharp for food-grade rPET. Regulation (EU) 2022/1616 and EFSA guidance make the quality of input material, process authorization, decontamination performance, and intended use central to food-contact recycling [6,7]. High collection alone cannot overcome colour mixing, non-PET contamination, viscosity loss, or processing rejects.

This paper develops a material-oriented assessment framework for DRS beverage packaging. It does not model household transport, route length, vehicle energy, or transport emissions. The system boundary begins at the return point and ends at the material-quality output gate. Within that boundary, the study combines return rate, stream quality, and processing or reuse yield for PET, aluminium, and reusable glass so that mass capture and high-value material recovery can be reported separately.

The same question becomes more practical in rural and small-settlement settings. In many villages, residents may not have a return point within the settlement and may keep empty containers at home until a trip to a nearby town. From a material point of view, the problem is not just the extra voyage. It is if storage time, residue, crushing, missing labels, or bad information on permitted formats lessens the chance of the container entering the designated material route. A smart-village perspective is relevant here because digital information, local service accessibility, and operator feedback can help preserve material quality before the return point is reached. Recent RURALSTRAT work in the Podlaskie region suggests technological, social, and governance tools for rural communities, including responses to transport difficulties and uneven digital competencies [8].

Reviews of deposit refund systems show that DRS can support high return rates and can be effective in closing beverage-container loops when infrastructure, deposits, labelling, consumer convenience, and operator coordination are aligned [9,10,11,12]. Nevertheless, many DRS performance indicators focus on collection or return percentages. For materials, this is incomplete because circularity depends on the material value of the returned stream, not only on the number of units returned.

The PET literature points to a set of bottlenecks that are directly relevant for a DRS stream: polymer identification, colour separation, labels and adhesives, caps and closures, moisture, intrinsic-viscosity retention, non-intentionally added substances, and decontamination capacity [13,14,15,16,17,18,19]. Mechanical recycling remains important for packaging circularity, but its output is sensitive to contamination and to the previous thermal and mechanical history of the polymer [17,18]. Studies on plastic contamination also show that small levels of non-PET polymers or metals can change the properties of reprocessed material [18,19].

Aluminium cans demand another kind of regulation. The metal can be recycled over and over again, but a specialized beverage can stream is still subject to scrap contamination, coating residues, dross losses, and alloy drift. Can-to-can or similar sheet production depends on sorting, decoating, remit yield, and final composition [20,21,22]. Even if the nominal return rate is high [21], dilution or down cycling may be needed if the stream is contaminated with other aluminium trash or with non-target elements.

Glass has to be handled differently again. In a reusable system, the preferred outcome is not cullet after each cycle but an intact bottle that passes inspection, washing, and refilling. Breakage, scuffing, bottle–pool mismatch, and washing rejection reduce the number of usable rotations [23,24,25]. For this reason, mass returned, mass recycled, and product reuse cannot be treated as a single recovery number.

The research gap addressed here is the absence of a compact, reproducible, and material-specific indicator that separates three different conditions: (i) whether a package is returned, (ii) whether the returned stream is of sufficient quality for a high-value route, and (iii) whether downstream reprocessing or reuse retains the material in the desired pathway. Existing policy reporting often treats collection as the main performance measure, while the material literature emphasizes that quality losses can occur after collection.

The novelty of this article is fivefold. First, it defines a high-quality recovery index (*HQR*) for DRS beverage packaging. Second, it applies the same mathematical structure to polymer, metal, and reusable glass pathways while preserving their different material-quality gates. Third, it introduces coefficient intervals and uncertainty envelopes rather than a single deterministic recovery value. Fourth, it incorporates a pilot PET stream-quality and processing-yield dataset to demonstrate how measured or operator-reported material-composition variables can be translated into *Q_PET_* and *Y_PET_*. Fifth, it specifies the operator measurements required to convert the model into a broader experimentally validated material-quality assessment for all DRS streams. The novelty is not the modelling of DRS logistics but the translation of material-quality degradation and processing or reuse losses into a material-specific high-value recovery indicator for PET, aluminium, and reusable glass.

## 2. Materials and Methods

The model covers material flows from the return point to the high-value output gate. Three streams are included: PET beverage bottles, where the desired output is food-grade rPET suitable for closed-loop beverage packaging; aluminium cans, where the desired output is alloy-compatible secondary aluminium for can-to-can or equivalent sheet production; and reusable glass bottles, where the desired output is a bottle retained for washing and refilling. The boundary excludes household travel, transport energy, vehicle emissions, and collection-route optimization [26,27,28].

Accordingly, the model boundary is a material-quality boundary rather than a deposit-system logistics boundary. The analysis begins after the packaging item has entered the return stream and evaluates whether the material can remain in the intended high-value material pathway.

**Figure 1:**
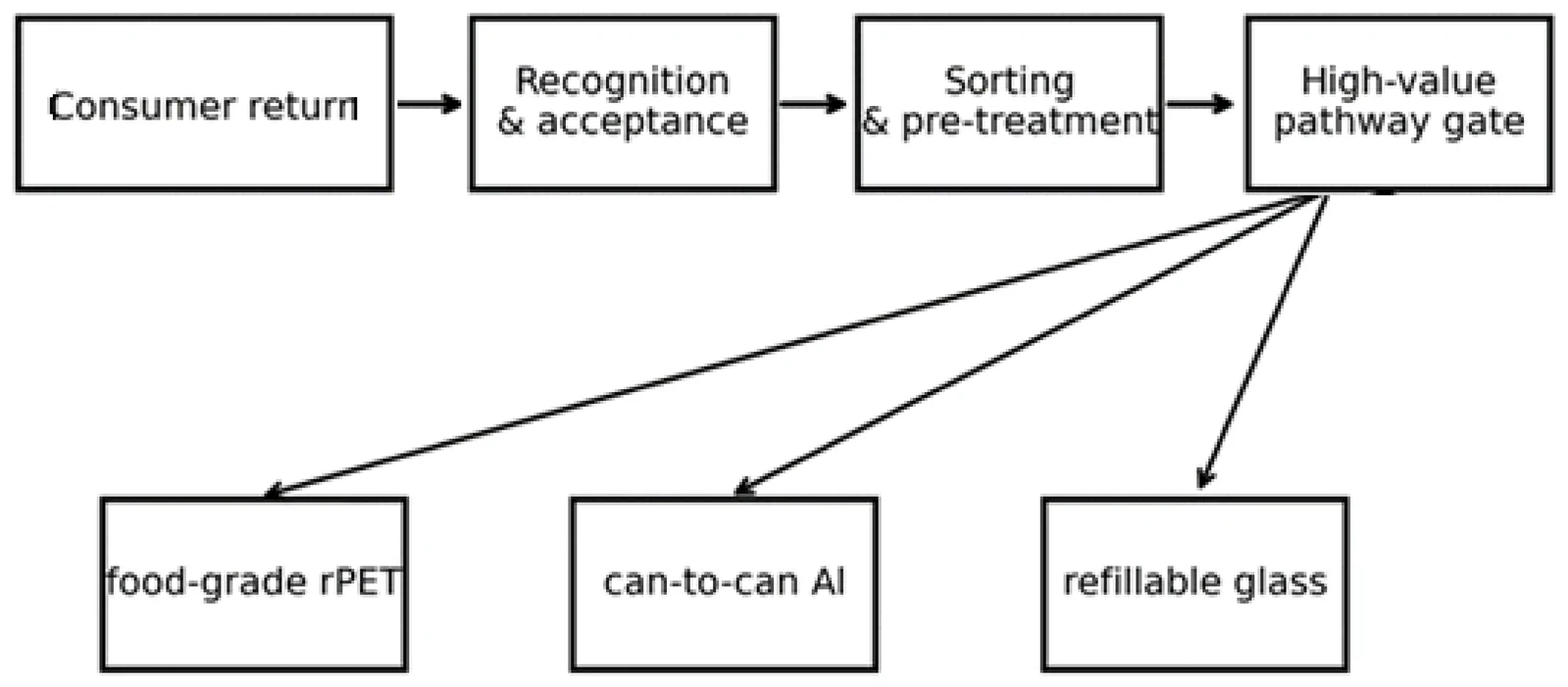
Material-loop system boundaries and quality gates considered in the material-circularity analysis. The boundaries begin at the return point and end at food-grade rPET, can-to-can aluminium, or refillable glass retention.

Figure 1 defines the system boundary and shows the quality gates used to separate return, material quality, and high-value recovery for the three beverage-packaging streams.

A project survey of 120 respondents was used only to define the early-stage return-rate scenario and to identify consumer behaviours that may influence material capture and return-point acceptance. The survey included questions on DRS awareness, return behaviour, return frequency, acceptable distance to return points, deposit acceptance, and perceived barriers. It was not treated as a nationally representative survey. Its role in the model is limited to defining a baseline value for R and qualitative constraints related to storage, residue, crushing, and acceptance at reverse-vending machines.

The survey-supported return scenario uses R = 0.50 because 50.0% of respondents declared that they already returned deposit packaging. A further 27.5% did not return packaging, and 22.5% planned to return it in the future. This baseline is compared with two policy-oriented scenarios: R = 0.77 and R = 0.90.

**Figure 2:**
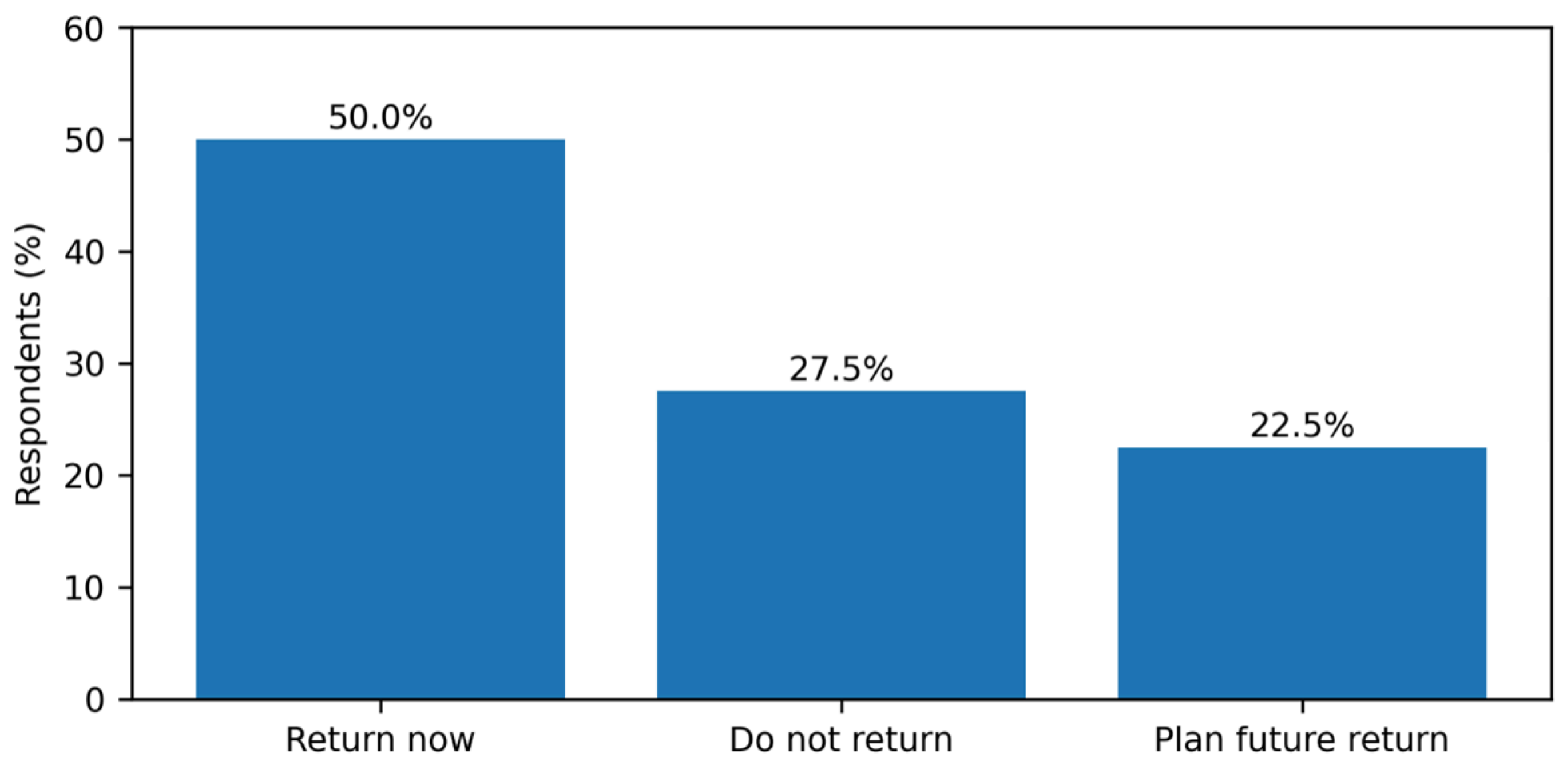
Survey-supported early-stage return behaviour used to define the baseline material-capture scenario.

Figure 2 summarizes the survey-supported early-stage return behaviour used as the baseline material-capture input in the model.

For each material stream m, high-quality recovery is calculated as an editable material-flow equation:

*HQR_m_* = *R* × *Q_m_* × *Y_m_*, (1)

where *HQR_m_* is the high-quality recovery index for material _m_, *R* is the return rate, *Q_m_* is the quality factor of the returned stream, and *Y_m_* is the reprocessing or reuse yield. All variables are dimensionless and range from 0 to 1. The equation can be applied to item counts or mass if the same basis is used consistently.

The circularity gap is defined as follows:

*CG_m_* = 1 − *HQR_m_*, (2)

The circularity gap represents the share of placed-on-market material that is not recovered as high-quality secondary material or retained as refillable packaging within the model boundary. To avoid overclaiming, *HQR_m_* is not interpreted as a national recycling rate. It is a quality-adjusted indicator for the target high-value route.

The *HQR* model should be interpreted as a sequential material-flow accounting indicator rather than as a mechanistic causal model. Its purpose is to estimate the fraction of placed-on-market packaging that remains in the target high-value circular pathway after three successive constraints have been applied: return, material-stream quality and downstream reprocessing or reuse yield. The multiplicative structure is used because a loss at any stage reduces the material quantity available for the next stage. The multiplicative form follows from a conditional material-flow logic. If one unit of packaging is placed on the market, only the fraction (R) is returned and becomes available for material-quality assessment. Of this returned fraction, only *Q_m_* remains suitable for the target high-value material route. Of the quality-accepted fraction, only *Y_m_* is retained after reprocessing or reuse. The final retained fraction is therefore the product of the three successive retention fractions. In this sense, *HQR_m_* = *R*\times *Q_m_*\times *Y_m_* is a mass-balance accounting expression for sequential losses, not an empirical regression equation and not a probabilistic independence assumption. A container that is not returned cannot enter the quality gate; a returned container that does not meet stream-quality requirements cannot enter the high-value pathway; and a material stream that passes the quality gate may still experience losses during washing, remelting, decontamination, inspection or refilling.

The model does not assume that *R*, *Q_m_* and *Y_m_* are statistically independent in real DRS operations. In practice, these variables may be correlated. For example, return-point accessibility, consumer instructions and reverse-vending reliability may influence both the return rate and the quality of the returned stream. Longer home storage before return may increase residue load, odour, crushing or label damage, thereby affecting both machine acceptance and downstream material quality. Similarly, better sorting infrastructure may improve *Q_m_* and also increase the effective yield *Y_m_*. Therefore, the three terms are treated as separable accounting components for transparency, not as independent causal drivers.

The equal multiplicative form also does not imply that *R*, *Q_m_* and *Y_m_* have equal practical weight in every system. Their relative importance depends on their observed values and variability. In an early-stage DRS, return rate may be the dominant limiting term. In a mature high-return system, material quality and processing yield may become more decisive. The model is therefore intended as a screening and reporting framework that can be updated with measured operator data, sensitivity analysis or material-specific weighting if future datasets show systematic correlations between return behaviour, stream quality and processing yield.

The model was strengthened by converting each quality factor into a set of measurable material variables. These variables are not all available in the current survey-supported dataset; they are specified as required operator or laboratory inputs for rigorous material-level validation. Table 1 lists the recommended variables for PET, aluminium, and reusable glass.

Bottle–pool mismatch denotes a returned reusable glass bottle that is intact but incompatible with the refillable bottle pool accepted by the washing and refilling operator because of differences in geometry, volume, neck finish, colour, marking, brand/pool ownership, crate compatibility or closure requirements.

For reusable glass, “bottle–pool mismatch” is defined as a return event in which a bottle is physically intact but does not belong to the refillable bottle pool accepted by the receiving washing and refilling system. A mismatch may result from incompatible bottle geometry, volume, neck finish, colour, embossing, permanent marking, brand-specific pool ownership, crate compatibility, closure type or inspection-code requirements [29,30,31]. Such a bottle may still enter a lower-value glass recycling route as cullet, but it cannot be counted as retained in the higher-value reusable bottle loop. In the *HQR* model, bottle–pool mismatch therefore reduces the reusable-glass quality factor (*Q_GLASS_*) or, where detected after washing-line inspection, the reuse-yield factor (*Y_GLASS_*).

The model combines two types of input. For PET, the generic coefficient envelope is supplemented with a pilot stream-quality and processing-yield dataset that demonstrates how measured or operator-reported material-composition variables can be translated into *Q_PET_* and *Y_PET_*. For aluminium and reusable glass, the coefficient envelope was informed by available member-state statistics, sectoral monitoring information and policy-relevant reporting on beverage packaging recovery, aluminium can recycling and reusable glass retention. These data do not represent plant-level measurements from one specific DRS operator; rather, they define a statistical-monitoring-data-informed screening range for material quality and yield.

For aluminium cans, the coefficient range reflects the generally high purity and recovery potential of dedicated used beverage can streams, while allowing for losses caused by ferrous and non-aluminium contamination, organic coating, decoating, dross formation, remelting yield and alloy-control constraints. For reusable glass, the coefficient range reflects the fact that a returned bottle remains in the higher-value reuse loop only if it passes bottle–pool compatibility, inspection, washing and refill-acceptance gates. Breakage, scuffing, incompatible formats and washing rejection therefore reduce the effective reuse yield.

The coefficients in Table 2 should therefore be interpreted as screening coefficients informed by statistical and sectoral monitoring data, not as arbitrary assumptions and not as direct plant-level measurements. In future validation, the aluminium coefficients should be replaced by operator-specific data on used beverage can purity, OES/XRF alloy composition, decoating loss, dross formation and remelted metal yield. The reusable glass coefficients should be replaced by operator data on bottle–pool mismatch, inspection rejection, washing rejection, breakage, scuffing and average refill rotations.

Although the aluminium and reusable glass coefficients were informed by member-state statistics, sectoral monitoring data and policy-reporting ranges, they should not be interpreted as direct measurements from one specific DRS operator. They were used to define a transparent screening envelope for the HQR model. Operator-level validation remains necessary to replace these ranges with site-specific values for alloy composition, remelting yield, bottle–pool mismatch, washing rejection, breakage and refill rotations.

A structured pilot PET stream-quality and processing-yield dataset was used to demonstrate how regional material-quality indicators can be translated into the *Q_PET_* and *Y_PET_* terms of the *HQR* model. The dataset compares Lithuania with sixteen Polish regions and includes PET stream purity, colour composition, non-PET impurity categories, residual moisture, organic and beverage residues, intrinsic viscosity, washed PET flake or pellet yield, and rejection after sorting and washing. These indicators were selected because they correspond to the PET quality and yield gates defined in Table 1.

In the pilot calculation, washed PET flake or pellet yield was used as a preliminary proxy for *Y_PET_*, because full-chain bottle-to-bottle operator data were not available. This proxy does not include all downstream losses during extrusion, melt filtration, decontamination, intrinsic-viscosity retention or restoration, pellet-quality control, food-contact compliance and final acceptance for beverage-bottle production. Therefore, the pilot *HQR_PET_* values should be interpreted as screening-level estimates and may represent upper-bound values relative to a complete food-grade rPET production chain.

The dataset was used as a pilot material-quality input rather than as a complete national inventory or a full industrial audit of PET recycling plants. The regional values should therefore be interpreted as screening-level material-quality indicators. They are suitable for demonstrating the model logic and for comparing how regional PET stream composition can affect *Q_PET_*, *Y_PET_*, and *HQR_PET_*, but they should not be interpreted as official national recycling statistics.

The available data are regional summary values. Raw batch-level measurements, sample counts, sample masses, sampling dates, replicate measurements, and laboratory measurement uncertainties were not available for all regions. For this reason, statistical uncertainty is not reported as a standard deviation or confidence intervals. Instead, uncertainty is represented by the regional minimum–maximum range and by the coefficient-envelope analysis used in the *HQR* model. In future validation, this dataset should be replaced or supplemented by operator-level and laboratory-level records, including the number and mass of PET samples, sampling period, sorting method, laboratory protocol for intrinsic viscosity, moisture determination, residue measurement, washing yield, and rejection logs. For the same reason, formal hypothesis tests between Lithuania and the Polish regional values were not performed. The comparison is descriptive and model-oriented; it is used to demonstrate how differences in PET purity, colour composition, impurity profile, IV and washed PET yield affect *Q_PET_*, *Y_PET_* and *HQR_PET_*, rather than to infer statistically significant national differences. Table 3 summarizes the regional-level pilot PET stream-quality and processing-yield indicators used to parameterize QPET and YPET in the HQR model.

Three return-rate scenarios were evaluated: survey-supported early stage (R = 0.50), EU interim target (R = 0.77), and mature high-return *DRS* (R = 0.90) (see Table 4). For each material and scenario, minimum, central, and maximum *HQR* values were calculated using Equation (1). The calculation uses only dimensionless parameters and can therefore be reproduced directly in a spreadsheet. 

The HQR intervals reported in this study are deterministic scenario envelopes, not statistical confidence intervals. They were obtained by applying lower, central and upper values of *Q_m_* and *Y_m_* in Equation (1). No probability distributions were assigned to the coefficients, and no inferential uncertainty was estimated because independent operator-level datasets and replicate measurements were not available for all material streams. The intervals should therefore be interpreted as sensitivity ranges showing how high-quality recovery changes under conservative, central and favourable material-quality and yield assumptions.

Because the purpose of the model is to support material-quality reporting rather than transport-energy accounting, rural accessibility was not converted into route length or vehicle emissions. Instead, the rural dimension was treated as an information and quality-control layer that can determine whether returned packaging reaches the correct material pathway. For rural settlements, the relevant operational variables include the availability of nearby return points, the current status of reverse-vending machines, the accepted packaging formats, rejection reasons, the storage time before return, the container condition, the residue level, crushing or label damage, and the share of packages that are transported to urban return points during other trips.

In a smart-village implementation, these variables could be collected through a mobile application, municipal information platform, or operator dashboard. Such a tool could inform residents where containers can be returned, whether a machine is active or full, which material fractions are currently accepted, and how containers should be stored to preserve PET recognition, colour separation, aluminium purity, and reusable glass refill suitability. The same system could provide anonymized quality feedback to operators, for example, the frequency of barcode failure, crushed-bottle rejection, non-PET contamination, residue-related rejection, bottle–pool mismatch, or refill rejection. These data would allow for the *HQR* model to be updated from coefficients to region-specific material-quality indicators, especially for rural areas where return behaviour and material storage conditions may differ from urban settings.

## 3. Results

The survey indicates that awareness and material capture are not the same. Although the underlying project dataset showed high awareness of the Polish *DRS*, only half of respondents declared that they already returned deposit packaging. From a material perspective, the unreturned share is not merely a behavioural loss; it is a direct feedstock loss before any recycling or reuse quality gate can operate.

Open-ended responses identified material-quality risks: the storage of empty containers in small dwellings, unpleasant odours from residues, the need to keep containers uncrushed and intact, reverse-vending machine failures, and the inconvenience of transporting many packages at once. These barriers can influence whether containers are returned, whether they are accepted by machines, and whether they enter the correct high-quality stream.

The regional-summary pilot PET dataset illustrates that returned PET streams can differ substantially in quality even before downstream food-contact assessment. Lithuania showed the most favourable PET stream composition, with 98.2% PET purity, 80.0% clear PET, 11.0% light-blue PET, and 1.8% total non-PET impurities. The Polish regional average was lower, with 94.3% PET purity, 72.7% clear PET, 10.6% light-blue PET, and approximately 5.7% total non-PET impurities. The dominant impurity categories in the Polish regional dataset were paper and label residues, HDPE, and PP, whereas PVC remained low in absolute terms but is still relevant because of its disproportionate effect on PET reprocessing quality. Because the dataset consists of regional summary values, these differences should be interpreted as screening-level evidence of material-quality variation rather than as statistically representative national estimates. No *p*-values are reported for this comparison because the dataset does not contain replicate batch-level observations or sample-specific uncertainty estimates. The numerical comparison is therefore limited to descriptive differences, regional ranges and their effect on the *HQR* model parameters.

The processing-quality indicators showed the same pattern. Lithuania had 0.55% residual moisture, 0.40% organic and beverage residues, an intrinsic viscosity of 0.74 dL/g, a washed PET flake or pellet yield of 84.5%, and a sorting/washing rejection rate of 15.5%. Across the Polish regions, the corresponding average values were approximately 0.90% residual moisture, 1.20% organic and beverage residues, 0.721 dL/g intrinsic viscosity, 80.3% washed PET yield, and 19.7% rejection after sorting and washing.

When PET purity and colour composition were combined, the estimated *Q_PET_* was approximately 0.894 for Lithuania and 0.786 for the Polish regional average. The available washed PET flake or pellet yield provided preliminary *Y_PET_* proxy values of 0.845 and 0.803, respectively. Because this proxy does not include all downstream bottle-to-bottle losses, the resulting *HQR_PET_* values should be interpreted as screening-level or upper-bound estimates rather than verified final food-grade rPET recovery rates.

Under the survey-supported R = 0.50 scenario, these pilot inputs gave an estimated *HQR_PET_* of approximately 37.8% for Lithuania and 31.6% for the Polish regional average. This result shows that nominal return rate alone can overestimate high-quality PET circularity when stream composition and processing yield are not reported separately.

Table 5 reports the central high-quality recovery index values for PET, aluminium, and reusable glass under the three return-rate scenarios using the generic coefficient envelope.

The deterministic interval results show how strongly quality and yield assumptions affect material circularity. These values are sensitivity ranges obtained from the lower, central and upper coefficient envelope; they should not be interpreted as confidence intervals or probability-based uncertainty estimates. Table 6 gives minimum, central, and maximum HQR values for each material. The uncertainty is largest for PET and reusable glass because their high-value pathways are more sensitive to quality gates: food-contact acceptability for PET and product-retention conditions for glass.

**Figure 3:**
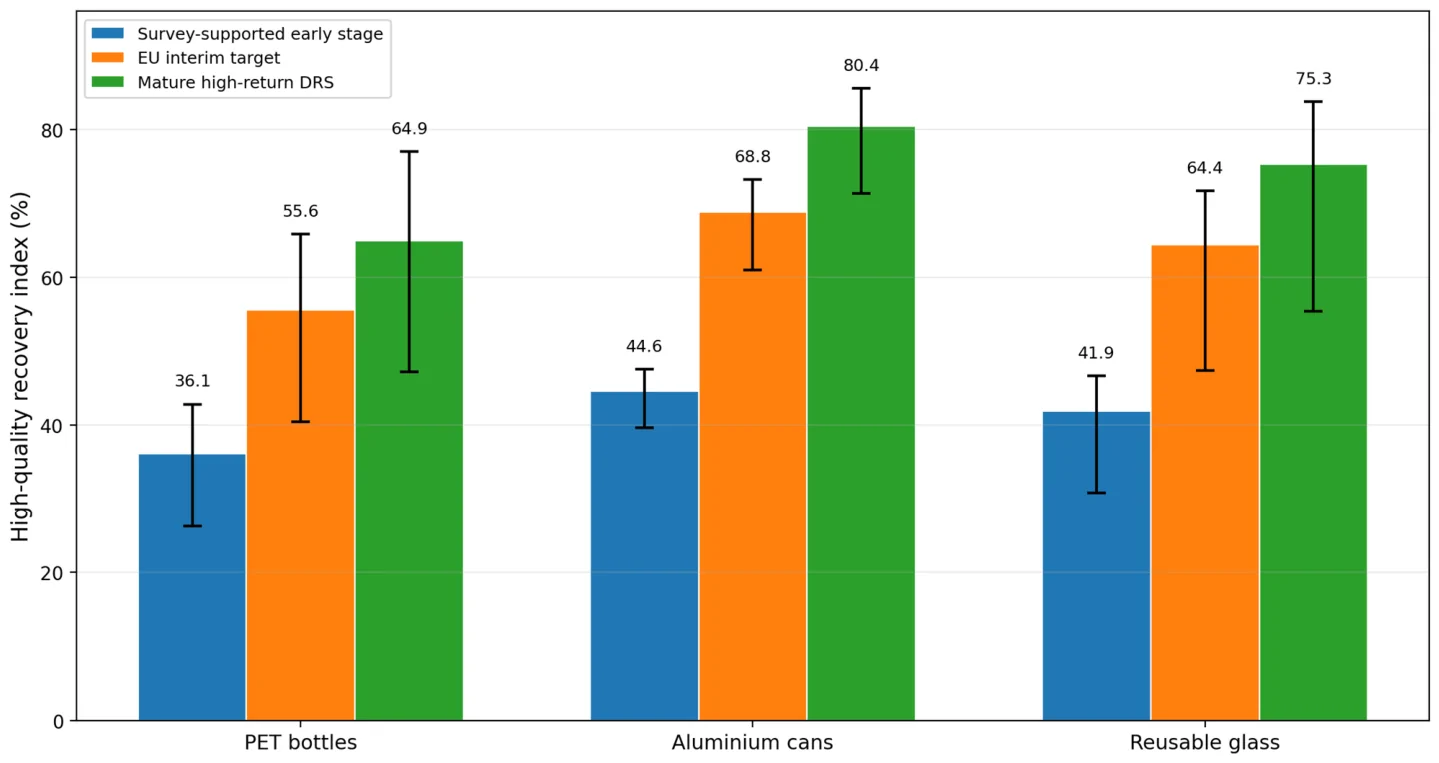
High-quality recovery intervals for PET, aluminium, and reusable glass under survey-supported, interim-target, and mature-DRS scenarios.

Figure 3 visualizes the HQR uncertainty intervals generated by the lower, central, and upper quality-yield coefficient envelopes.

Across the generic scenarios, PET remains the most constrained stream. This should not be read as evidence that PET recycling is ineffective; it indicates that the food-grade bottle-to-bottle route is more selective than a general PET recovery route. A returned bottle may be acceptable for a lower-value outlet while still being unsuitable for beverage-grade rPET. Reporting should therefore separate the PET collected, PET recycled, and PET returned to food-contact bottle applications.

Aluminium has the highest central *HQR* in the selected scenarios because a well-separated used beverage can stream combines high purity with a high metal yield. That result still depends on material control. Ferrous contamination, non-can aluminium scrap, coating losses, dross, and alloy composition can all reduce the suitability of the recovered metal for can-to-can or equivalent sheet production.

Reusable glass is governed by product retention rather than by a simple recycling yield. A bottle that survives handling and passes washing remains in the refill system; a broken, heavily scuffed, or incompatible bottle leaves the higher-value reuse route and may become cullet. Reuse reporting should, therefore, include rotations, washing rejects, and breakage, not only the tonnage of glass returned.

To avoid presenting the material-specific differences only qualitatively, the central HQR results were further decomposed into model-based loss components. For each material stream, the placed-on-market amount was divided into unreturned material ((1 − *R*)), material-quality gate loss (*R*(1 − *Q_m_*)), downstream yield loss (*RQ*_m_(1 − *Y_m_*)), and high-quality recovery (*RQ_m_Y_m_*). This decomposition shows where the main losses occur within the *HQR* structure and provides a quantitative basis for comparing PET, aluminium and reusable glass.

Under the mature high-return scenario (R = 0.90), PET bottles show 10.0% unreturned material, 10.8% quality-gate loss, 14.3% downstream yield loss and 64.9% high-quality recovery. Aluminium cans show 10.0% unreturned material, 4.5% quality-gate loss, 5.1% downstream yield loss and 80.4% high-quality recovery. Reusable glass shows 10.0% unreturned material, 6.3% quality-gate loss, 8.4% downstream yield loss and 75.3% high-quality recovery. The comparison confirms that PET is the most constrained stream because both material quality and downstream yield losses are higher than for aluminium and reusable glass.

**Figure 4:**
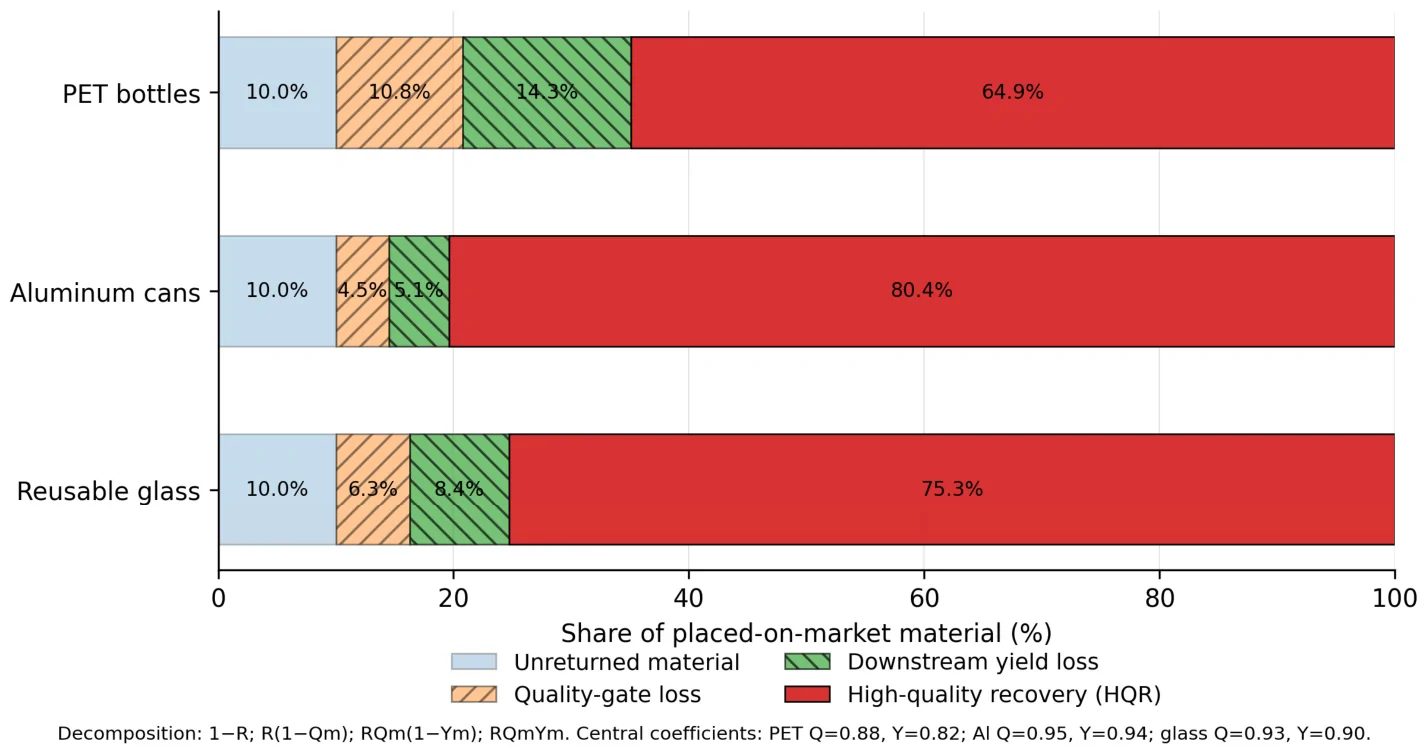
Quantitative decomposition of the central HQR result under the mature high-return DRS scenario (R = 0.90).

Figure 4 presents this quantitative decomposition of the central HQR result under the mature high-return scenario.

The numerical values used to construct Figure 4 are reported in Table 7 to provide a transparent quantitative decomposition of the central *HQR* result and to show the relative contribution of non-return, material-quality loss, downstream yield loss and high-quality recovery for each packaging stream.

Table 7 confirms that PET has the largest combined quality and yield losses among the three streams. Under the same return-rate scenario, PET loses 25.1% of placed-on-market material after return through quality and yield constraints, compared with 9.6% for aluminium and 14.7% for reusable glass. This quantitative decomposition supports the conclusion that high return rates alone are not sufficient to ensure high-quality circularity, especially for food-grade PET pathways.

## 4. Discussion

The main result is that *DRS* performance changes once it is expressed as a quality-adjusted material outcome. Return rate is needed, but it is only the first term in the chain. The *HQR* index indicates losses at two further points: the quality of the returning stream and the yield of the chosen reprocessing or reuse pathway. It links policy objectives with physical limits, such as PET purity and appropriateness for food contact, control of aluminium alloy, and the continuation of refillable glass bottles [32,33,34].

The interpretation of the three *HQR* terms requires caution. The model separates *R*, *Q_m_* and *Y_m_* to make reporting transparent, but this separation should not be read as proof that the variables are independent in practice. In a real DRS, the same operational decisions may affect more than one term. For example, improved return-point availability may increase *R*, reduce home storage time and improve *Q_m_*, while better sorting technology may improve both *Q_m_* and *Y_m_*. The model therefore provides a structured accounting framework rather than a full causal representation of the DRS. Its advantage is that it makes the location of losses visible and allows for future operator data to replace generic coefficients or to test correlations between the terms.

The calculation also indicates why headline return rates can be misleading. A 90% collection level does not mean that 90% of the packaging becomes high-quality recovered material, unless both *Q_m_* and *Y_m_* are close to one. On the other hand, even a pristine stream cannot compensate for poor capture. The value of the simple multiplicative equation is that it obliges operators to report capture, quality, and yield as separate quantities.

**Figure 5:**
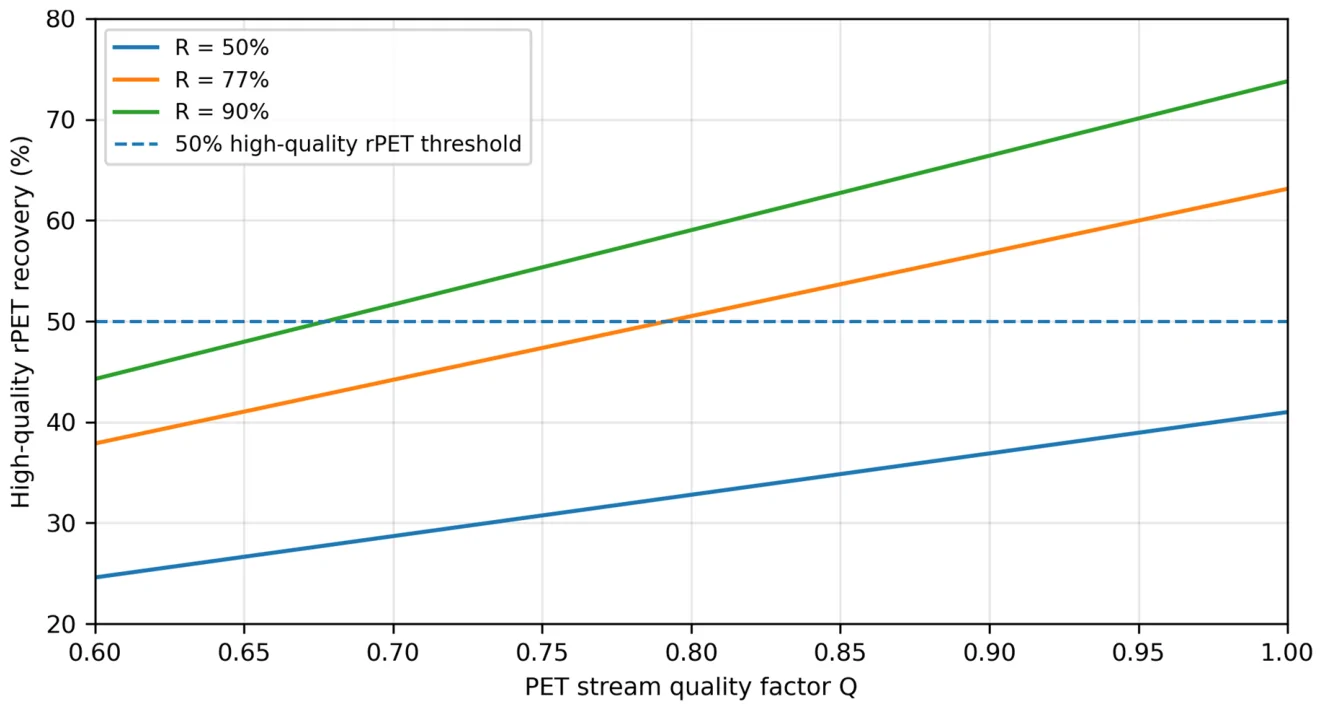
PET high-quality recovery threshold as a function of return rate and stream-quality factor. At low return rates, even a perfect quality factor cannot reach the 50% high-quality rPET threshold; at high return rates, material-quality losses remain decisive.

PET is the most quality-sensitive stream in this study; therefore, the discussion graph focuses on the relationship between return rate and PET stream quality. Figure 5 indicates how much high-quality rPET is recovered based on *Q_PET_* at three different return rates, while keeping *Y_PET_* at the average processing yield. The 50% horizontal line is used as a practical threshold to show when at least half of placed-on-market PET becomes high-quality rPET within the model boundary.

Figure 5 presents a threshold analysis for PET, showing how return rate and stream quality jointly determine whether high-quality rPET recovery can exceed 50%.

The threshold plot is useful for three reasons. At R = 50%, the 50% high-quality rPET line cannot be reached even when *Q_PET_* is near 1.0, because too little material is entering the system. At R = 77%, the threshold becomes attainable, but only if the PET stream remains sufficiently clean and colour suitable. At R = 90%, the system has a wider margin, although contamination, colour mixing, damaged containers, or food-contact rejection can still keep high-quality rPET recovery below the desired level. Return-point coverage and PET quality control therefore have to be improved together.

The PET results should be read in the context of food-contact recycled plastics. Under Regulation (EU) 2022/1616 and EFSA guidance, a post-consumer PET process intended for food-contact use must rely on authorized recycling, controlled input, suitable pre-processing, effective decontamination, and a defined final use [6,7]. These are not formalities; they decide whether the recovered polymer can return to beverage packaging [35,36,37].

This is illustrated for the pilot PET dataset. Lithuania shows high purity of PET, a high clear/light-blue fraction, low non-PET contamination, low residual moisture, low organic residue, IV = 0.74 dL/g, and an 84.5% washed PET yield. It has poorer PET purity, greater non-PET contaminants, and a lower cleaned yield than the Polish regional average. Assuming the same R = 0.50, these inputs produce *HQR_PET_* values of around 37.8% and 31.6% for Lithuania and the Polish regional average, respectively. The difference is in the composition of colour, contaminant profile, and the processing yield, not in the nominal return rate.

The nature of the impurity fraction is also important. The greatest non-PET fractions found in the Polish regional dataset are paper and label residues, HDPE and PP. Although the fraction of PVC is modest, it is considered a serious pollutant because even small quantities can degrade the quality of PET reprocessing. Therefore, PET reporting should be based both on the overall non-PET contamination and on the distribution of contaminant kinds, rather than a single purity percentage.

Another example of a realistic compromise is the requirement of containers to remain intact. Reverse-vending machines can more easily identify and authenticate intact bottles, and intact bottles remain traceable through their labels or barcodes. However, whole bottles occupy more room in residences and are more prone to cause smell concerns when residues remain. Crushing decreases, the volume but can damage the label, barcode, or bottle shape. Hence, the impact of volume-reduction strategies on recognition, sorting, and downstream rPET quality [19] as well as convenience should be assessed.

Aluminium is often touted as a material that can be recycled over and over again, with industry sources emphasizing that the metal does not lose its inherent quality during recycling [22]. This means that not every returned can gets a new can sheet automatically. The recycling process for used beverage cans still requires separating the can stream from steel, non-target aluminium, and other pollutants; removing coatings; controlling dross formation; and ensuring that the final alloy composition meets the specifications. For this reason, scrap upgrading, impurity removal, and remelting are considered core quality procedures in reviews of aluminium recycling [20,21].

For the HQR model, this means that *Q_Al_* and *Y_Al_* should be populated with sorting-purity, alloy-composition, and remelting-yield data whenever such data are available. A high can return rate without information on alloy compatibility is not enough to support a can-to-can circularity claim.

Reusable glass follows a product-retention logic. After return, the bottle is inspected, washed, and either kept in the refill pool or removed from it. Breakage, severe scuffing, incompatible formats, and washing rejection reduce the reuse yield. In this context, bottle–pool mismatch refers to an otherwise returnable glass bottle that cannot be accepted by the specific refill system because its geometry, volume, neck finish, colour, permanent marking, brand or pool ownership, crate compatibility or closure requirements differ from those used by the washing and refilling operator. Broken bottles may still remain within a glass-material loop as cullet, but they no longer deliver the higher-value function of a refillable package.

A reusable glass indicator should, therefore, measure rotations and reasons for rejection in addition to mass returned. If the standard bottle pools, inspection regulations, or washing processes are insufficient, a system may collect a substantial volume of glass but perform poorly as a reuse system. The same structure of *HQR* can be altered to make the refill rotations the functional basis of the computation when industrial data are available.

The pilot PET results also make the case for searching outside the large urban return networks. A hamlet home might retain bottles for days or weeks and return them only on a visit to a town with a reverse-vending machine. This rural path is obscured if you simply measure performance at the city return point. Bottles can retain residues, acquire odours, be compressed to conserve space, lose barcode readability, or be contaminated with non-target packaging during storage. The deposit may be the same, yet these conditions affect recognition, sorting, and high-value material gates.

So, for the material-flow analysis, a smart village layer is not an add-on but can help to safeguard the quality of the material stream. A rural DRS needs to have clear, up-to-date information on where containers can be returned, if machines are working, what formats are accepted, and how PET, aluminium, and reusable glass should be prepared. This function might be helped by mobile applications, municipal information boards, or operator dashboards that would reduce failed returns and discourage storage or preparation procedures that impair material quality.

The same information layer could also supply operators with material data. Rejection causes, machine failures, non-PET contamination, residue-related rejects, barcode problems, bottle–pool mismatch and refill rejection can all be mapped to the *Q* and *Y* terms in the *HQR* model. This is particularly important where the system appears to work at a city return point but performs less well in surrounding villages. The practical question is whether sending bottles from villages to urban machines is materially sensible: does it preserve high-value PET, aluminium, and glass pathways, or would local/mobile return options provide better capture and better quality? The RURALSTRAT smart-village framework offers a useful policy context for this kind of information-based *DRS* material-quality layer [8].

This manuscript was deliberately framed to avoid overlap with energy-logistics analysis. No route length, household vehicle use, fuel consumption, electricity demand, or transport emissions were calculated. The only behavioural input is the return share used to define material capture. All results concern material quality and high-value recovery after the return point. This separation makes the materials’ contribution independent: it addresses what fraction of returned packaging can become high-quality secondary material or retained reusable packaging.

The present application should therefore be viewed as a structured screening exercise rather than as independent validation of the model. The *HQR* equation identifies where data should be collected and how return rate, material quality and processing or reuse yield can be reported separately. Its validation requires external operator datasets that were not available in the present study. Such datasets should include return-point rejection causes, sorting-plant purity measurements, PET washing and decontamination yields, aluminium alloy composition and remelting yields, and reusable glass inspection, washing, breakage and refill-rotation records.

The following are practical recommendations for operator reporting. The HQR model should be used as a reporting structure rather than as a substitute for measured operator data. DRS operators should report return rate and high-quality recovery as separate indicators for each material stream. For PET, beverage-grade rPET yield should be differentiated from lower-value PET recovery routes. Reverse-vending rejection causes should be recorded, including damaged labels, crushed bottles, barcode failure, residues and wrong-material inputs. Aluminium reporting should include can-stream purity, alloy composition and remelted metal yield. Reusable glass reporting should include washing rejection, breakage, bottle–pool mismatch and refill rotations. Anonymised operator datasets and calculation files should be made available to enable independent validation of material-quality claims.

The new model has obvious limitations. The numbers for PET composition and processing yield are inputs to the pilot, not a nationwide industrial inventory. They demonstrate how composition and processing measures can be incorporated into *Q_PET_* and *Y_PET_* but should not be regarded as official Lithuanian or Polish DRS statistics. Coefficients for aluminium and reusable glass are scenario-based, as operator data on alloy composition, remelting yield, bottle–pool mismatch, washing rejection, and refill rotations were not available. The survey is likewise small and non-representative; hence, its return-rate value is utilized just as an early-stage scenario.

The model was not independently validated with external operator datasets. Consequently, the calculated *HQR* values should be interpreted as scenario-based screening outputs rather than verified predictions of DRS performance. The uncertainty intervals are deterministic coefficient envelopes generated by interval arithmetic. They are not statistical confidence intervals and were not derived from probability distributions. Future validation should combine the *HQR* framework with independent plant-level datasets and replicate measurements so that confidence intervals, probability distributions or Monte Carlo uncertainty analysis can be introduced.

The main limitation of this study is that the PET stream-quality and processing-yield dataset is available only as a regional-summary pilot dataset. It demonstrates how PET purity, colour composition, impurity profile, intrinsic viscosity, residual moisture and washed PET yield can be incorporated into *Q_PET_* and *Y_PET_*, but it does not provide raw batch-level measurements for all regions. Therefore, sample counts, sample masses, sampling dates, replicate measurements, laboratory uncertainty and confidence intervals could not be reported. The results should not be interpreted as official Lithuanian or Polish DRS statistics or as a complete industrial inventory. They are screening-level inputs used to demonstrate the *HQR* model and to identify which PET quality variables should be measured systematically in future operator validation. Consequently, the Lithuania–Polish regional comparison should not be interpreted as a hypothesis-tested difference. It is a descriptive screening comparison designed to show how regional material-quality indicators affect the HQR calculation. Future studies should collect replicate PET samples from defined time periods and processing batches so that statistical tests, confidence intervals and measurement-error propagation can be applied.

A further limitation concerns the PET yield term. In the pilot calculation, *Y_PET_* was approximated using washed PET flake or pellet yield because full-chain bottle-to-bottle yield data were not available. This approximation does not capture all downstream losses during extrusion, melt filtration, decontamination, IV retention or restoration, pellet-quality control, food-contact compliance and final bottle-grade acceptance. As a result, the reported *HQR_PET_* values may overestimate final beverage-grade rPET recovery.

A more rigorous validation would be to combine the *HQR* model with plant and lab data for all three streams. For PET, the pilot dataset should be enriched with measures of sorting purity, colour fractions, IV, moisture, non-PET contamination, decontamination performance, and food-contact rPET pellet yield. For aluminium, future work should include used beverage can purity, OES alloy analysis, decoating loss, dross, and remelted metal yield. The data needed for reusable glass include breakage, washing rejection, scuffing, pool compatibility, and refill rotations. A model with those inputs would evolve from a structured assessment framework to a predictive tool for DRS material performance.

## 5. Conclusions

This study proposed a quality-gated material-flow framework for evaluating high-value circularity in deposit return systems. The central contribution is not the calculation of return rate alone but the separation of three sequential conditions: whether packaging is returned, whether the returned stream retains sufficient material quality, and whether the downstream process retains the material in the intended high-value pathway. This distinction is important because the same nominal return rate can lead to different circularity outcomes for PET bottles, aluminium cans and reusable glass bottles.

The *HQR* framework should be interpreted as a transparent screening and reporting tool rather than as a fully validated predictive model. It separates *R*, *Q_m_* and *Y_m_* for reporting purposes, while recognising that these terms may be correlated in real DRS operation. The deterministic *HQR* envelopes are sensitivity ranges, not confidence intervals. They identify where losses may occur and which material-quality variables should be measured before operator-level validation is possible.

The pilot PET results demonstrate how regional stream-quality indicators can be translated into model inputs. Lithuania showed higher PET purity, a higher clear/light-blue fraction, lower non-PET contamination, lower residue and moisture levels, a higher IV and a higher washed PET yield than the Polish regional average. However, the calculated (HQR_{PET}) values should be treated as screening-level and potentially upper-bound estimates because the pilot *Y_PET_* term is based on washed PET flake or pellet yield, not on full-chain bottle-to-bottle food-grade rPET yield.

The quantitative decomposition of the mature high-return scenario showed that PET has the largest combined quality and yield losses among the three analysed streams. Under *R* = 0.90, PET loses 25.1% of placed-on-market material after return through quality and downstream yield constraints, compared with 9.6% for aluminium and 14.7% for reusable glass. This confirms that high return rates alone are not sufficient to demonstrate high-quality circularity, especially for food-grade PET pathways.

A practical implication of the study is that DRS reporting should move beyond returned mass. Operators should report material-specific purity, colour fractions, impurity profiles, rejection causes, processing yields, alloy compatibility, washing rejection, breakage and refill rotations. These variables would allow for generic *Q_m_* and *Y_m_* coefficients to be replaced by measured operator data.

The smart-village information tool proposed in this study provides a possible extension of the *HQR* framework for rural and small-settlement contexts. Such a tool could inform residents about active return points, accepted packaging formats and correct container preparation, while also collecting material-quality feedback for operators. In *HQR* terms, this information layer could help reduce losses associated with incorrect storage, residues, crushing, barcode failure, non-PET contamination, bottle–pool mismatch and refill rejection. Therefore, the smart-village component is not only a communication tool but also a potential source of material-quality data for improving *Q_m_* and *Y_m_* estimates in rural DRS implementation.

Future research should focus on operator-level validation. For PET, the washed-flake yield proxy should be replaced by cumulative bottle-to-bottle yield data covering sorting, washing, extrusion, melt filtration, decontamination, IV retention or restoration, pellet-quality control and final food-contact acceptance. For aluminium, future datasets should include used beverage can purity, alloy composition, decoating loss, dross formation and remelted metal yield. For reusable glass, future work should include bottle–pool mismatch, washing rejection, breakage, scuffing and refill-rotation records. Future studies should also test correlations between *R*, *Q_m_* and *Y_m_*, introduce probability-based uncertainty analysis when replicate data are available, and pilot a smart-village information tool that links return-point status, rejection causes and storage-related quality risks directly to *HQR* reporting.

Future research will be conducted on the data collected from real DRS processes in various localizations, including rural areas which substantially differ from densely habituated urbanized areas. Also, future research will take into account various logistic schemes, which might differ in energy requirement. Consequently, the energetic optimization of the DRS system will be analysed.

## Figures and Tables

**Table 1:** Material-specific quality gates and recommended validation variables for future operator or laboratory measurement.

Material Stream	High-Value Target	Quality Gate (*Q_m_*) Should Include	Yield Gate (*Y_m_*) Should Include	Suggested Validation Measurements
PET and bottles	Food-grade rPET and bottle-to-bottle use	Candrect PET identification; clear/light-blue fraction; low PVC, HDPE, PP, metal and paper contamination; readable label/barcode; low residue and moisture; and compliance with authorized input stream.	Flake washing yield; extrusion yield; decontamination efficiency; intrinsic-viscosity retention; and rejecting ion after food-contact assessment.	NIR sorting and purity; colour fraction; PVC/HDPE fraction; moisture; residue mass; intrinsic viscosity; GC-MS challenge test or surrogate decontamination results; and rPET pellet yield.
Aluminium cans	Can-to-can or equivalent aluminium and sheet	Used beverage can purity; low ferrous and non-aluminium contamination; alloy compatibility; and reduced organic coating and residue load.	Decoating loss; remeltinging yield; dross formation; and final alloy composition, within specification.	Manual/NIR/eddy-current sorting purity; OES alloy composition; Fe, Mg, Mn and Si levels; coating burn-off loss; dross mass; and remelted metal yield.
Reusable glass	Bottle retained for refill cycle	Correct bottle pool, defined as compatibility with the accepted refillable bottle system in terms of geometry, volume, neck finish, colour, permanent marking, brand/pool ownership, crate compatibility and closure requirements; intact geometry; readable marking; low chipping/scuffing; and no incompatible closures or foreign glass.	Inspection pass rate; washing retention; breakage and loss; refill acceptance; and average rotations.	Breakage rate; inspection rejection; washing rejection; bottle–pool mismatch rate; scuffing score; closure or neck damage; number of refill rotations; and final discard reason.

**Table 2:** Statistical-monitoring-data-informed coefficient envelope used for uncertainty analysis. Values are screening coefficients derived from available member-state, sectoral and policy-monitoring information; they are not plant-level measurements from a single DRS operator.

Material	*Q_min_*	*Q_central_*	*Q_max_*	*Y_min_*	*Y_central_*	*Y_max_*	Main Interpretation
PET bottles	0.75	0.88	0.95	0.70	0.82	0.90	Quality and yield are sensitive to contamination, colour composition, IV loss, residue load, washing losses and food-contact acceptance. The PET range is supported by the pilot PET dataset and by food-contact rPET quality constraints.
Aluminium cans	0.90	0.95	0.98	0.88	0.94	0.97	The range reflects statistical and sectoral evidence that dedicated used beverage can streams can reach high purity and high metal yield, while still requiring control of non-aluminium contamination, coating losses, dross formation, remelting yield and alloy compatibility.
Reusable glass	0.82	0.93	0.97	0.75	0.90	0.96	The range reflects monitoring-based uncertainty in maintaining bottles within the higher-value refill loop. Retention depends on intact bottles, bottle–pool compatibility, inspection pass rate, washing rejection, breakage, scuffing and refill acceptance.

**Table 3:** Regional-summary of the pilot PET stream-quality and processing-yield dataset used to parameterize *Q_PET_* and *Y_PET_*. Values are screening-level regional indicators; raw batch-level sample uncertainty was not available.

Dataset	PET Purity, %	Clear PET, %	Light-Blue PET, %	Non-PET Impurities, %	Dominant Impurity Profile, %	IV, dL/g,	Washed PET Yield, %	Estimated *Q_PET_*	*Y_PET_*	*HQR_PET_* at R = 0.50, %
Lithuania	98.2	80.0	11.0	1.8	PVC, 0.05; HDPE, 0.55; PP, 0.45; metals, 0.15; paper/labels, 0.60	0.740	84.5	0.894	0.845	37.8
Polish regions, mean	94.3	72.7	10.6	5.7	PVC, 0.16; HDPE, 1.64; PP, 1.34; metals, 0.53; paper/labels, 2.00	0.721	80.3	0.786	0.803	31.6
Polish regions, range	92.5–95.8	68–77	10–11	4.2–7.5	PVC, 0.10–0.23; HDPE, 1.25–2.15; PP, 1.00–1.75; metals, 0.40–0.72; paper/labels, 1.45–2.65	0.700–0.740	77.3–82.8	approx. 0.72–0.84	0.773–0.828	not applicable

Note: PET purity, colour composition and impurity fractions are used as material-quality indicators for *Q_PET_*, while washed PET flake or pellet yield is used as the processing-yield indicator for *Y_PET_*. The dataset is reported at the regional-summary level; therefore, uncertainty is expressed through regional ranges rather than through sample-level confidence intervals.

**Table 4:** Return-rate scenarios applied in the quality-gated model.

Scenario	Return Rate, R	Purpose in the Article
Survey-supported early stage	0.50	Baseline derived from the project survey.
EU interim target	0.77	Policy-oriented reference from the SUP separate-collection target.
Mature high-return DRS	0.90	High-performance DRS reference aligned with the 2029 target.

**Table 5:** Central high-quality recovery index values calculated with Equation (1) using the generic coefficient envelope.

Material	R = 0.50	R = 0.77	R = 0.90	Interpretation
PET bottles	36.1%	55.6%	64.9%	Lowest central HQR due to food-contact and contamination gates.
Aluminium cans	44.6%	68.8%	80.4%	Highest central HQR because purity and remelting yield are comparatively favourable in dedicated streams.
Reusable glass	41.9%	64.4%	75.3%	Intermediate central HQR; losses arise mainly from breakage and inspection rejection.

**Table 6:** Deterministic HQR sensitivity envelope. Minimum and maximum values combine the return-rate scenario with lower and upper quality/yield coefficients. The intervals are not confidence intervals and do not represent probability distributions.

Scenario	Material	*HQR_min_*	*HQR_central_*	*HQR_max_*	Circularity Gap at Central *HQR*
Survey-supported early stage	PET bottles	26.2%	36.1%	42.8%	63.9%
Survey-supported early stage	Aluminium cans	39.6%	44.6%	47.5%	55.4%
Survey-supported early stage	Reusable glass	30.8%	41.9%	46.6%	58.1%
EU interim target	PET bottles	40.4%	55.6%	65.8%	44.4%
EU interim target	Aluminium cans	61.0%	68.8%	73.2%	31.2%
EU interim target	Reusable glass	47.4%	64.4%	71.7%	35.6%
Mature high-return DRS	PET bottles	47.2%	64.9%	77.0%	35.1%
Mature high-return DRS	Aluminium cans	71.3%	80.4%	85.6%	19.6%
Mature high-return DRS	Reusable glass	55.4%	75.3%	83.8%	24.7%

**Table 7:** Quantitative decomposition of the central HQR result under the mature high-return DRS scenario (R = 0.90).

Material Stream	Unreturned Material, %	Quality-Gate Loss, %	Downstream Yield Loss, %	HQR, %
PET bottles	10.0	10.8	14.3	64.9
Aluminium cans	10.0	4.5	5.1	80.4
Reusable glass	10.0	6.3	8.4	75.3

## Data Availability

The original contributions presented in this study are included in the article. Further inquiries can be directed to the corresponding authors.

## Abbreviations

The following abbreviations are used in this manuscript:

*DRS*	Deposit Return System
PET	Polyethylene Terephthalate
rPET	Recycled Polyethylene Terephthalate
*HQR*	High-Quality Recovery Index
*CG*	Circularity Gap
R	Return Rate
*Q*	Material-Quality Factor
*Y*	Reprocessing or Reuse Yield
RVM	Reverse Vending Machine
IV	Intrinsic Viscosity
NIR	Near-Infrared Sorting
GC-MS	Gas Chromatography–Mass Spectrometry
OES	Optical Emission Spectroscopy
HDPE	High-Density Polyethylene
PP	Polypropylene
PVC	Polyvinyl Chloride
EFSA	European Food Safety Authority
SUP	Single-Use Plastics
EU	European Union
